# The Impacts of Surface Microchannels on the Transport Properties of Porous Fibrous Media Using Stochastic Pore Network Modeling

**DOI:** 10.3390/ma14247546

**Published:** 2021-12-08

**Authors:** Xiang Huang, Wei Zhou, Daxiang Deng, Bin Liu, Kaiyong Jiang

**Affiliations:** 1 Fujian Key Laboratory of Special Energy Manufacturing, Xiamen Key Laboratory of Digital Vision Measurement, Department of Mechanical Engineering and Automation, Huaqiao University, Xiamen 361021, China; mold_bin@hqu.edu.cn (B.L.); jiangky@hqu.edu.cn (K.J.); 2 Department of Mechanical & Electrical Engineering, Xiamen University, Xiamen 361005, China; weizhou@xmu.edu.cn; 3 School of Mechanical Engineering and Automation, Harbin Institute of Technology, Shenzhen 518055, China; dengdaxiang@hit.edu.cn

**Keywords:** random pore network modeling, tailored structure, transport property

## Abstract

A stochastic pore network modeling method with tailored structures is proposed to investigate the impacts of surface microchannels on the transport properties of porous fibrous media. Firstly, we simplify the original pore network extracted from the 3D images. Secondly, a repeat sampling strategy is applied during the stochastic modeling of the porous structure at the macroscale while honoring the structural property of the original network. Thirdly, the microchannel is added as a spherical chain and replaces the overlapped elements of the original network. Finally, we verify our model via a comparison of the structure and flow properties. The results show that the microchannel increases the permeability of flow both in the directions parallel and vertical to the microchannel direction. The microchannel plays as the highway for the pass of reactants while the rest of the smaller pore size provides higher resistance for better catalyst support, and the propagation path in the network with microchannels is more even and predictable. This work indicates that our modeling framework is a promising methodology for the design optimization of cross-scale porous structures.

## 1. Introduction

The understanding of the structure–performance relationship of porous media and the optimization of the microstructure is the key to the improvement of its performance applied in various engineering applications involving gas diffusion layers, catalyst support layers, and capillary wicks. The transport property is strongly impacted by the pore phase morphology. Considering the application of fibrous porous material as catalyst support layers, a small pore size attributed to a larger specific surface area leads to an enhanced catalyst loading and reaction, while a larger pore size or specially processed structures, such as grooved microchannels, can provide a highway for flow so that the reactants can be removed in time. In a word, the presence of different pore sizes results in an ideal solution satisfying both properties dominated at different length scales. Nowadays, effects in developing such a multiscale, or in other words, a hierarchy structure for optimized engineered material, has gained considerable attention [1]. For example, P. Trogadas et al. [2] proposed lung-inspired fractal microchannel flow fields applied in polymer electrolyte fuel cells (PEFC) and outperformed the conventional serpentine flow field due to the more uniform reactant distribution. Alternatively, Y. Zhang et al. [3] achieved the desired uniformity in current density by tailoring the porosity variation in the gas diffusion layer (GDL) using a gradient configuration. Briefly, the microchannels [4,5,6] and pore structures [7,8,9,10] demonstrate significant influences on the mass transport properties and have been extensively explored.

**Figure 1:**
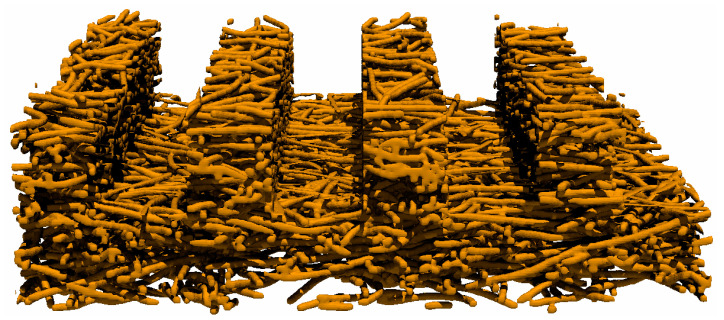
Illustration of a combination of straight line microchannels above the fibrous porous structure.

These works further inspire the idea of the combination of microchannels and pore structures (Figure 1). Fabricating microchannels on porous structures was proposed [11,12,13] and applied in a methanol steam reforming (MSR) as catalyst support layers, and enhanced hydrogen production was obtained comparing the ones without microchannels. However, on the one hand, experimental measurements for obtaining physical properties could be hard to conduct due to a small pore size. On the other hand, numerical modeling is also difficult since the computational capacity is insufficient to handle a heterogeneous structure. Fortunately, we can apply pore networking as an alternate. Pore network modeling [14] is a kind of pore-scale model that accounts for the true geometrical heterogeneity but simplified geometrical details of the porous material at the pore scale. Despite the simplification, its validation is testified through a comparison with the Lattice Boltzmann method in our earlier efforts [15,16]. The pore network can be extracted from 3D images [17,18] using imaging processing, but the extraction process is nontrivial, let alone hierarchical materials with multi-scale structures. To this end, Sadeghi et al. [19] constructed a 2D regular lattice network consisting of nanopores and macropores were later added into it with its overlapping nanopores removed. This hierarchical pore network architecture was further extended to 3D cases by Moghaddam et al. [20] However, both of the pore networks [19,20] are constructed on regular 2D or 3D lattices, which deviates from the extracted ones in nature. Jiang et al. [21] proposed an approach to generate from an extracted network a stochastic network of arbitrary size. Pores were randomly located in the given network domain and connected by throats according to their correlations. The flow properties involving capillary pressure, absolute and relative permeability were preserved in the stochastic network.

In this work, to explore the impacts of microchannels on the transport properties of porous fibrous materials, we generate a random network to construct the porous structure at the macroscale. The pore and the throat radius, and the coordination numbers (the number of throats connected to a pore) are assigned to the elements (pores and throats) of the random network via repeatedly sampling from the element data of the extracted network of the 3D images. Microchannels are then modeled as spherical pore chains and added to the random network. The structure and transport properties of the pore network with and without microchannels are discussed.

## 2. Materials and Methods

### 2.1. Pore Network Characterization

#### 2.1.1. Network Extraction

**Figure 2:**
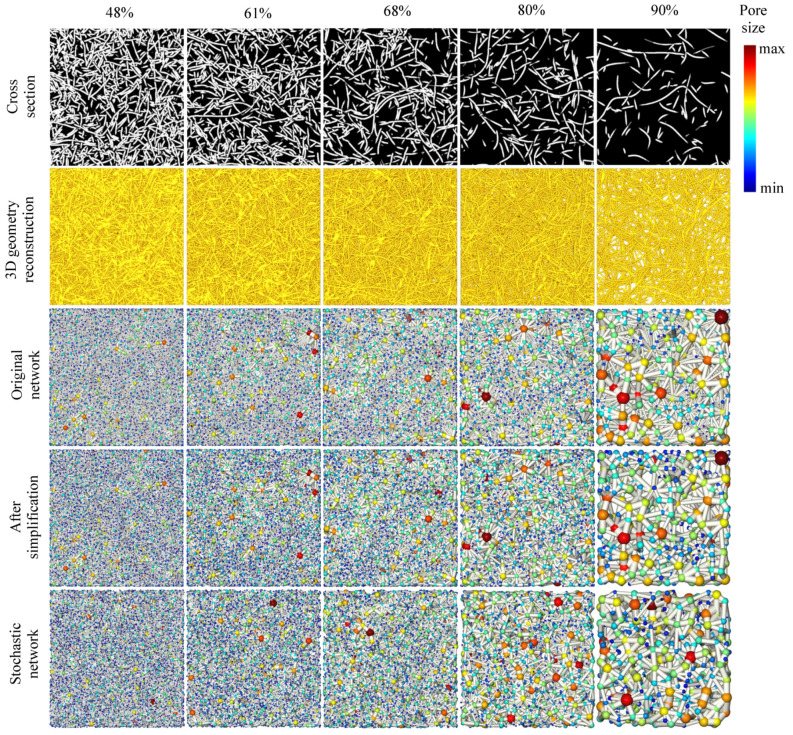
In the column direction from left to right: fiber mat of porosities of 48%, 61%, 68%, 80%, and 90%; in the row direction from top to bottom: the cross-section image, the 3D geometry reconstruction, the extracted pore network of the void space, the network after simplification, and the stochastic network. The 3D image is of 400 × 400 × 100 (x × y × z) pixel resolution, with 5 pixels in the fiber’s width.

The network extraction from 3D images can be achieved using different methods, involving skeletonization [21] and watershed segmentation [18] methods. The structures of the extracted network vary using different methods [22] and result in the different customization of the parameter towards fitting to the transport properties. The material we discussed is a kind of fibrous porous material composed of curved copper fibers of a mean diameter of 100 μm, and these fibers are compressed in the mold and sintered to form a connected fibrous porous structure with a mean pore diameter of about 300 μm [11]; therefore, the diffusion phenomena occur at the meso, and macroscale and are driven by the concentration gradient (molecular diffusion) rather than the collision of gas molecules with pore walls (Knudsen diffusion). In our former works, we applied the watershed segmentation method to obtain the network structure from the 3D images of porous fibrous materials and effectively predicted transport properties (invasion percolation and diffusion) by characterizing the throat radius using the area-equivalent radius [15,16]. To investigate the impact of porosity on the pore network, 3D images of fibrous porous material of different porosities (48%, 61%, 68%, 80%, and 90%) are generated using our earlier methods [23]. Pore networks are then extracted from the 3D images and demonstrated in Figure 2. The dimension is 400 × 400 × 100 (x × y × z) pixels for each 3D image, with a resolution of 5 pixels in fiber width.

#### 2.1.2. Original Network Simplification

##### Filter Throat Using a Threshold Conductive Value

In the original networks, determined by the number of the adjacent pores, pores may have very high coordination numbers (the number of throats connected to a pore), i.e., over a hundred. The connection of pores according to the coordination number is a nontrivial task during the virtual network generation process, and thus it is beneficial to simplify the network at an early stage. The reduction of coordination number is conducted through choosing a threshold value of the hydraulic conductance of the throat, throats of conductive values less than the threshold one only have little impact on the transport property can be removed. The hydraulic conductance *g* (Equation (1)) [14] of a cylindrical shaped throat is defined to be proportional to the fourth power of the throat diameter *D_t_* and inversely proportional to the throat length *L*_t_. *μ* is the viscosity of the fluid.

(1) $$g=\frac{\pi }{128\mu }\left(\frac{D_{t}^{4}}{L_{t}}\right)$$

**Figure 3:**
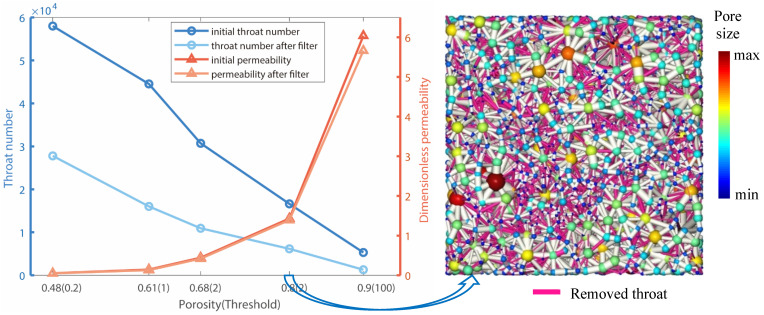
Impact of threshold value on throat filter. The threshold values applied for fibrous porous structures with porosities of 48%, 61%, 68%, 80%, and 90% are 0.2, 1, 2, 5, and 100, respectively. The effects are promising since a large portion of throats can be removed with little impact on the permeability values.

Figure 3 shows the impact of threshold values on the throat filter. The threshold values applied for fibrous porous structures with porosities of 48%, 61%, 68%, 80%, and 90% are 0.2, 1, 2, 5, and 100, respectively. These threshold values are chosen via repeat trial. The effect is promising since a large portion of throats can be removed with little impact on the permeability values.

##### Throat Merge

**Figure 4:**
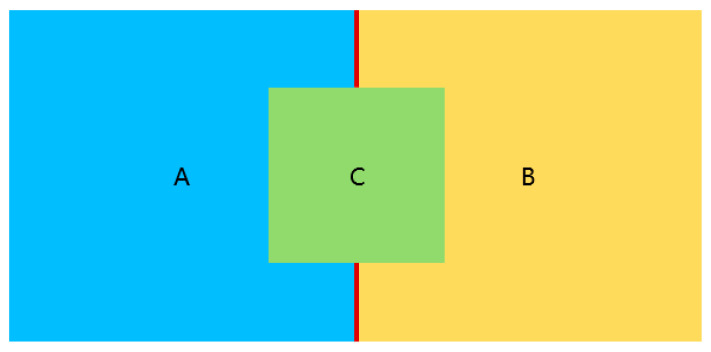
2D illustration of the multi throat connection between a pore-pore pair. Square A, B, and C indicate the space division after the watershed segmentation, each square is later identified as a pore cell, pore cells ‘A’ and ‘B’ shares more than one border shown as red lines, which are identified as throats.

Two pores in the initial network after watershed segmentation may be connected by more than one throat. We use the 2D scenario (Figure 4) for illustration, squares A, B, and C indicate the space division after the watershed segmentation, each square is then identified as a pore cell. It shows that pore cells A and B share more than one borders, where each border is later considered as a throat. In our stochastic pore network, we preserve only one throat in each pore-to-pore pair; therefore, an equivalent cylinder throat is constructed considering the physical properties contributed by all of the connecting throats, that is, assuming there are *n* throats *t_i_* of diameters *d_i_* (*I* = 1,…, *n*) in a pore–pore pair, according to Equation (1), the diameter *d*’ of the merged throat with the equivalent hydraulic conductance contributed by all of the *n* throats is deduced as Equation (2):

(2) $$d\prime ={\left(\sum_{1}^{n}d_{i}{}^{4}\right)}^{\frac{1}{4}}\;(i=\;1,\ldots ,n)$$

The area-equivalent throat radius is processed as well.

##### Prune Dead-End Branch of the Network

The dead-end branch is located where the pore owns only one throat. Since the mass transport is blocked in the dead ends, these branches can be removed without affecting the transport property.

Table 1 summarizes the number of reduced elements at each simplification process. Note that a pore will be isolated and then removed if all of its neighbor throats are removed. The simplified networks are demonstrated in Figure 2 (bottom row).

Once network simplification is finished, the structure information of each network element is recorded and statistically described. The structure information for each pore includes radius and coordination number, and for each throat includes radius, equivalent radius, and length.

### 2.2. Stochastic Network Generation

#### 2.2.1. Basic Idea

Once the simplification work is done, a statistical study of the structural property of the pore network can be performed, including the distributions of pore radius, throat radius, throat lengths, and the coordination number relationships. Based on the statistics data of the simplified network, we can generate the equivalent stochastic one of arbitrary size. We directly generate the network element data with geometry and topology property honoring its essence by simply sampling from the original statistical data without mathematically solving the correlations (regression or conditional probability distribution suggested in Ref. [21]) between the properties of elements, i.e., the coordination number of a certain pore is assigned using the one from the sampled pore element rather than determined by a mathematical description of their correlation.

#### 2.2.2. Generate Pores

**Figure 5:**
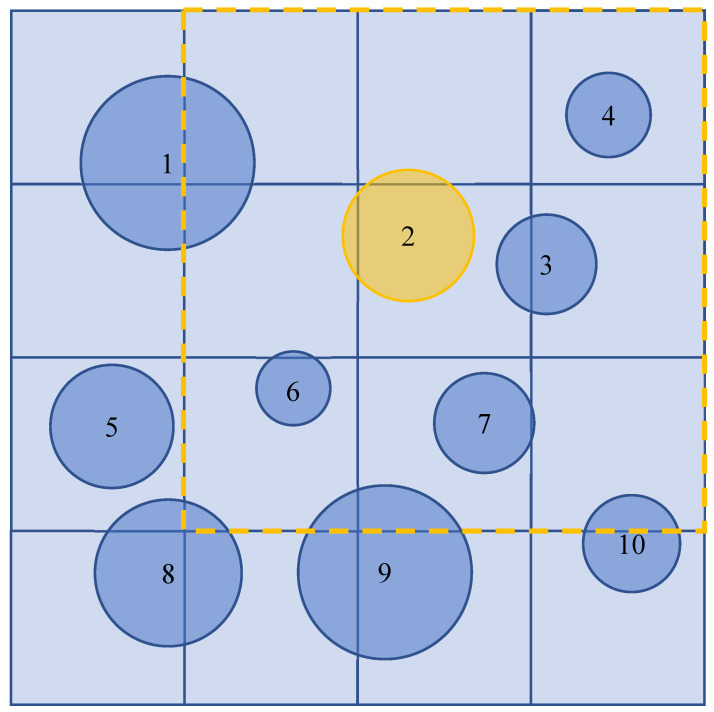
Illustration of overlapping detection in the 2D example. Pore 2 need to check the pores in the neighbor cubic blocks surrounded by the highlighted dash lines, that is, pore 4, 3, 6, and 7.

To generate a stochastic network of certain porosity. First, according to the target modeling domain size, we generated the required number of spherical shaped pores while avoiding overlapping between them. The positions of the pores are randomly chosen. The structural property of each pore is determined via randomly sampling from the statistics data, involving radius and coordination number. The computation capacity for overlapping detection between pores run out quickly at higher pore number, to overcome it, the space-time trade-off is applied, that is, to reduce the time consumed in performing the task via providing more space used to store extra data. Using a 2D example for clarity (Figure 5), the modeling domain is divided into cubic blocks of equal size. The side length of each block needs to be no less than the diameter of the maximal pore in the network. Then the overlapping detection of a pore in the whole system can be limited to the detection of pores located in its neighbor 3 × 3 blocks (similarly extended to 3 × 3 × 3 blocks for 3D example).

#### 2.2.3. Generate Throats

##### Throat Location

**Figure 6:**
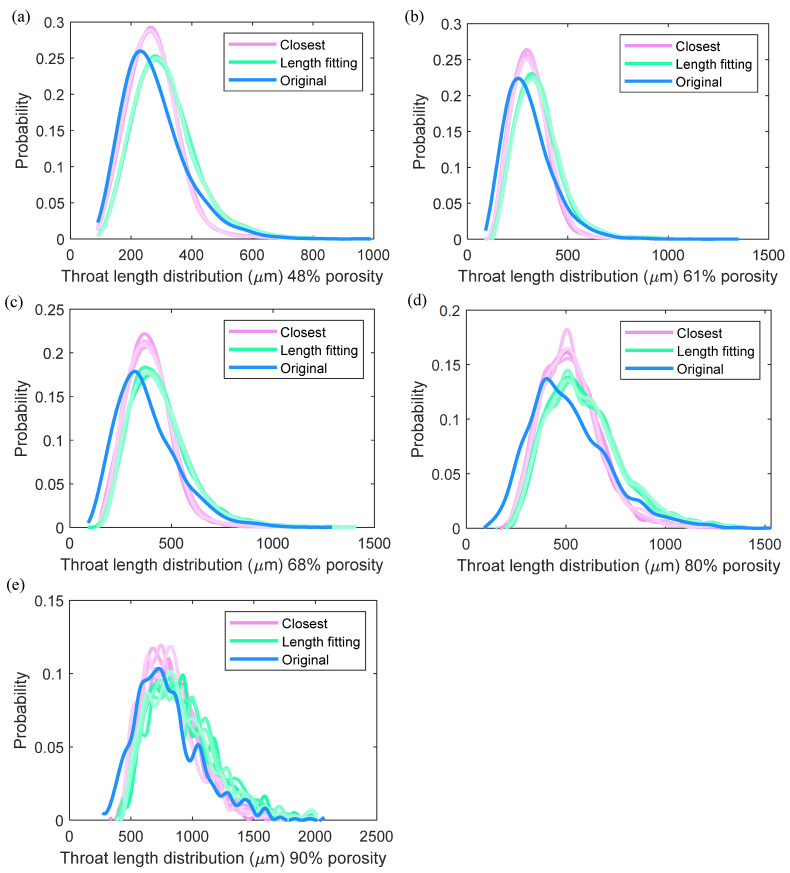
Comparison of throat length distributions using different generation rules (method I and method II) at different porosities (**a**) 48%, (**b**) 61%, (**c**) 68%, (**d**) 80%, and (**e**) 90%. The distributions generated using the closest neighbor rule (method II) and the length fitting rule (method Ⅰ) are close to the original one, but neither of them can perfectly match the original one. For each method, five stochastic realizations are shown.

A strategy is required to determine the generation sequence and which pore to pore pair to choose for throat generation. For the first issue, since larger pore sizes dominate the mass transport, we give high priority to large pores by sorting the pores according to their radius. The throats are then generated for each pore following the sorted pore sequence, while to the best honoring the coordination number of each pore. For the second issue, to determine which pore to connected with, Jiang [21] and Chalendar [24], using the distribution of the throat length as the principle to direct the pore–pore pair selection (Method I), that is, the pore to pore pair of the closet distance length to the target throat length following the throat length distribution is chosen. However, it can be pretty far off the target length among the available pore candidates (pores of distances less than the longest throat length, and meanwhile can still provide throat links (the number of throats of the candidate pore has not reached its coordination number yet)), especially towards the end of the process, when not many available pores can still provide the connection. Alternately, we can also generate a throat by simply following the closest neighbor rule (Method II). That is, the throat is generated by choosing from the available pores of the smallest distance to the current connecting pore. Figure 6 shows the comparison of the throat length distributions of the two methods to the original one. It can be detected that the distributions generated using the length fitting rule and the closest neighbor rule are close to the original one, but neither of them can perfectly match the original one, especially the underpredicted values for the low throat length region, which could be attributed to a specific spatial distribution of the pore location in the original network, rather than randomly located as assumed in our modeling. However, we detect that the mean throat length of method II is closer to the original one, we thus chose the closest neighbor rule for throat generation. Finally, note that towards the end of the generation process, some pores may fail into awkward situations where no more throat links can be generated from the available candidate pores, in this case, if the number of the already generated links is more than two, which is assumed as the minimal coordination number for the network connectivity, the throat linking process for this pore is terminated at the price of disobeying its coordination number, if less than two, we assign two pores in its neighbor region to retain the network connectivity, at the price of disobeying and exceeding the coordination numbers of the two neighbor pores.

##### Throat Properties

The throat properties of radius and equivalent radius can be assigned afterward. We repeatedly sample from the throat dataset until the required throat number is reached. We compute a weight for each throat as the smallest radius of its two connecting pores. We assign the sampled throat radius accordingly, with the largest throat radius and its equivalent radius assigned to the throat of the largest weight.

#### 2.2.4. Generate Boundary Throats

We generate the boundary throat to connect the boundaries (inlet and outlet surfaces) and their nearby pores within a certain distance from the boundaries. The distance value is determined by honoring the number of pores connected to the boundaries in the original network.

**Figure 7:**
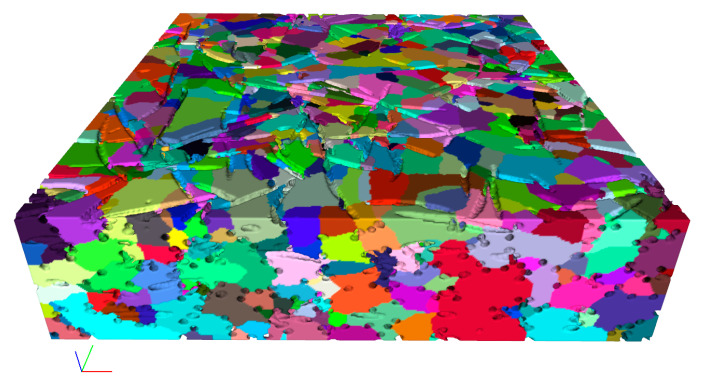
The boundary faces are divided into many boundary cells as the byproducts of watershed segmentation in the 3D domain.

In the original network, during watershed segmentation, the 2D boundary surface of the 3D domain is divided into many boundary cells (Figure 7), with each 2D boundary cell belonging to a certain 3D pore cell. For the boundary throat, its radius in Equation (1) is assigned according to the shape of the 2D boundary cell, and the distance in Equation (1) is assigned as the distance between the boundary cell mass center and its corresponding pore cell mass center. However, in the stochastic network, in the absence of the watershed segmentation effect, the boundary throat radius is assigned equal to the connecting pore’s value. The boundary throat length is assigned as the distance between the pore center and the boundary surface, and this assumption might lead to the deviation of the permeability calculation as will be discussed in the following. Finally, the generated stochastic networks are shown at the bottom of Figure 1. Good similarity can be observed between the stochastic and the original network structure.

### 2.3. Microchannel Modeling

**Figure 8:**
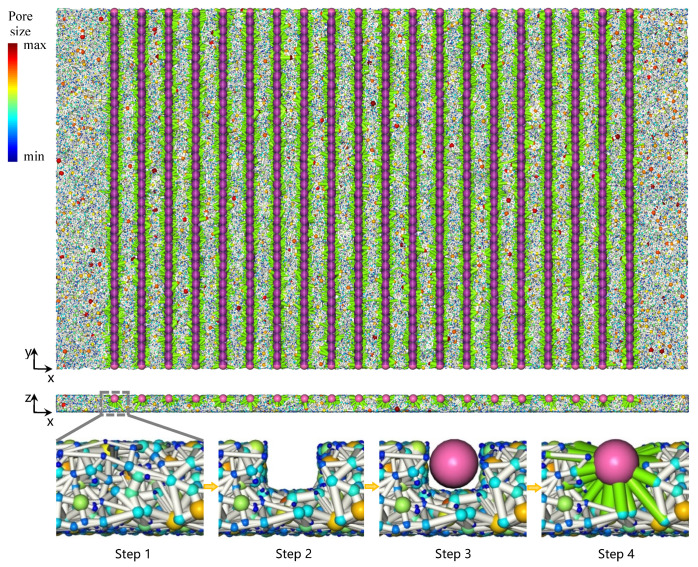
Stochastic pore network modeling of 80% porosity porous fibrous material with microchannels. Step 1: the initial 80% porosity stochastic network. Step 2: dig out elements in the microchannels, the hanging throats are not shown. Step 3: add channel pore chains shown in purple. Step 4: generate channel throat between adjacent channel pores shown in transparent purple to avoid covering the channel pore, the hanging throat is reconnected as being shown in green.

We utilize the stochastic network generation algorithm to investigate the material reported in Ref. [13], where 80% porosity fibrous porous material of macroscopic size of 70 × 40 × 2 mm^3^ (x × y × z) is processed with parallel rectangular microchannels. The cross-section size of each microchannel is 1 × 1 mm^2^, and the adjacent microchannel spacing is 2 mm. There are mainly four steps to generate its network representation as shown in Figure 8. In step 1, we generated the stochastic network of the macroscopic size. Step 2, we remove pores in the microchannels, some throats will be removed if their two connecting pores are both removed, some throats will turn hanging with only one connecting pore left. Step 3, we refill the microchannels with channel pores of 1 mm diameter. Step 4, we recover the network connectivity, including generating channel throats between each channel pore and reconnecting the hanging throat in step 2 to the nearby channel pore.

The structural properties of the added throats are then assigned. For the channel throat, its radius is assigned equal to the radius of the channel pore (500 μm) and its equivalent radius is calculated according to the channel’s cubic cross-section area. The channel throat length is assigned equal to the distance between the two adjacent channel pore centers. For the hanging throat, since there is no obvious clue towards its property, its radius is somewhat arbitrarily assigned equal to the smaller connecting pore, and its equivalent radius is arbitrarily assigned equal to the mean value of its two connecting pore radiuses. The generated pore network with surface microchannel is shown in Figure 6. This network is later applied in the water transport simulation.

## 3. Results and Discussion

### 3.1. Validation of Stochastic Network

We validate the stochastic network by assessing if its structural and transport properties of the same size as the original one are faithfully reproduced. For each porosity, we generate 5 stochastic networks of size equal to the original one. The throat length distribution is discussed above. For other properties we used the repeatedly sampling strategy for pore element characterization. The distributions of pore radius, throat radius, and equivalent radius are almost identical to the original ones. The distribution of the coordination number and the correlation between pore radius and coordination number also match the original data well, despite some coordination discord occurring in the throat generation process.

**Figure 9:**
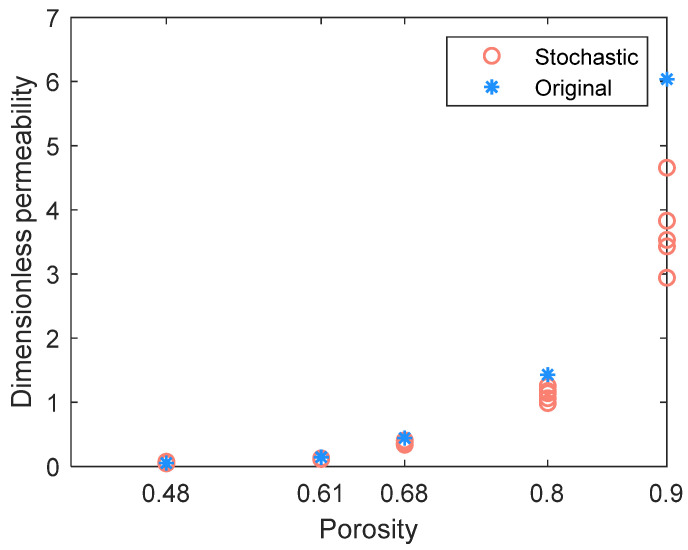
Dimensionless permeabilities in the in-plane direction of the original network and stochastic realizations. Five stochastic realizations are generated for each porosity.

**Figure 10:**
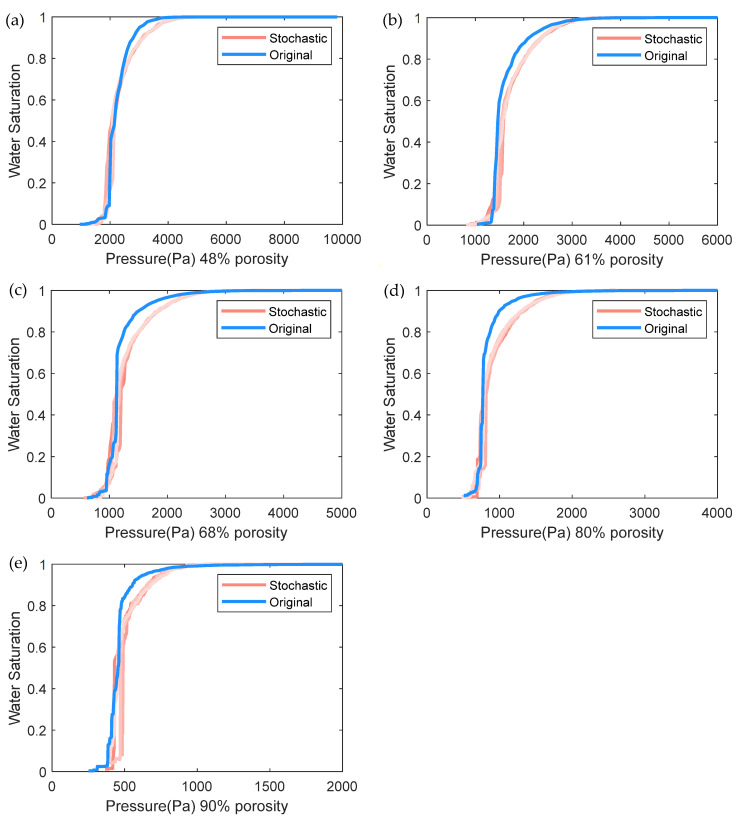
Capillary pressure curves of the original and stochastic pore network. Five stochastic realizations are generated for each porosity (**a**) 48%, (**b**) 61%, (**c**) 68%, (**d**) 80%, and (**e**) 90%.

We then compared the dimensionless permeability (permeability divided by the square of fiber radius, Figure 9) and the capillary pressure curve (Figure 10) in the in-plane direction. The permeability value is obtained using Darcy’s law with the pressure of each pore in the network obtained according to mass conservation. For implementation details, readers can refer to [14]. Figure 9 shows that the stochastic permeability values tend to be underestimated as the porosity increases, compared to the original ones. Note that in the stochastic networks, in the absence of the watershed segmentation effect, the properties of boundary elements (radius and length in Equation (1)) are estimated according to the properties of the nearby pores rather than the properties of the segmented boundary elements in the original network. This leads to the deviation of the calculated conductivity values (Equation (1)) at the boundaries, and finally the underpredicted permeability, especially for the 90% porosity as shown in Figure 9. Note that the permeability value is calculated according to the total flux at the boundary side (either inlet or outlet), and thus the calculated value is deeply dependent on the hydraulic conductive value (Equation (1)) of each boundary note. To better reproduce the permeability value, a more sophisticated understanding of the parameters in Equation (1) is required, including the distribution of the boundary note’s diameter, the distance to its neighbor pore, and their combination. Anyhow, the predicted permeabilities of different porosities are quite distinct, the deviations of porosities less than 80% are acceptable, and the deviation from the original results will not affect the following discussion of the effect of microchannel on the flow properties for a certain porosity.

We then compared the capillary pressure curves of the stochastic and original networks in Figure 9. The capillary pressure *P_c_* is calculated according to the Young–Laplace equation:

(3) $$P_{c}=-\frac{2\gamma \cos\theta }{R}$$

where *γ* = 0.072 Nm^−1^ is the water/air surface tension. *θ* is the contact angle and is assumed as 180°, and *R* is the throat radius and assigned as the area-equivalent radius in this work. The capillary pressure vs saturation curve can be obtained after applying the invasion percolation algorithm, which simulates the invasion of the nonwetting phase (water) in the wetting phase (air) saturated domain usually conducted at room temperature. For implementation details of the invasion percolation algorithm, readers can refer to [14]. We can see in Figure 9 that for each porosity, the sharp increasing region of curves matches well, with some deviation at the higher saturation section, and overall good agreements are achieved. In addition, the sharp increasing region left shifts as the porosity increases. This is as expected, since the steep region corresponds to the rapid propagation of the invasion fluid (water) when the capillary pressure exceeds this particular throat entry pressure, which is correlated to the throat radius and increases as the porosity increases, and the capillary pressure is inversely proportional to the throat radius.

### 3.2. Impact of Microchannel on the Transport Property

#### 3.2.1. Throat Radius–Saturation Relationship

**Figure 11:**
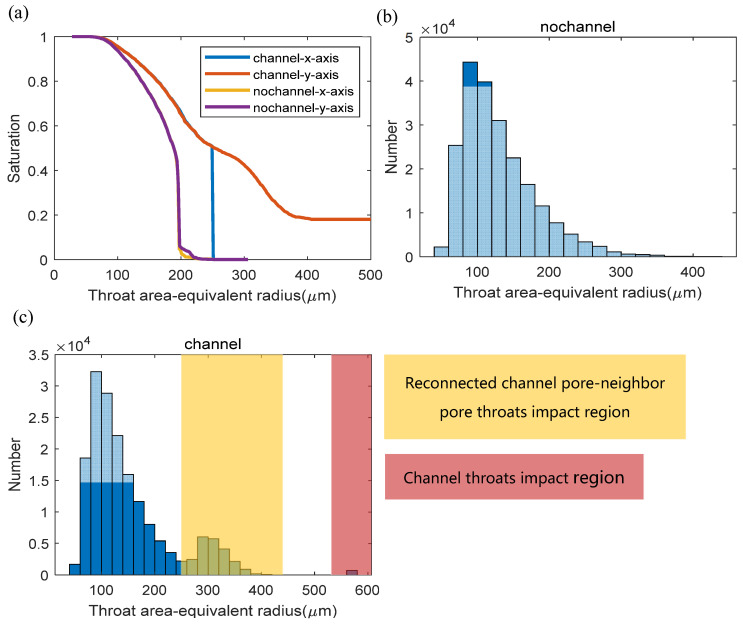
(**a**) Throat area-equivalent radius and saturation relationship curves, and throat area-equivalent radius distributions of pore network without (**b**) and with (**c**) microchannels.

We first investigate the capillary pressure curves with and without microchannels (Figure 11). In the model, the straight microchannels are arranged along the *y*-axis, and the flow both along the *x*-axis and *y*-axis is predicted. We use the throat area-equivalent radius-saturation curves instead of the traditional capillary pressure–saturation curves, in association with the distribution of the throat radius, to more directly indicate the structure-correlated transport property. We can see in Figure 11a that the curves of the model without microchannels (so-called no-channel) in the *x-* and *y*-axis directions are quite identical. The steep region of the curve indicates the rapid propagation of the invasion fluid and occurs at the throat area-equivalent radius of about 200 μm. It indicates that a large portion of the void phase is dominated by pores connected by at least one throat of an area equivalent radius higher than 200 μm and can be invaded and completely connected from the inlet surface.

The curves of the micro-channeled network show different shapes. Compared with Figure 11b, we can find two distinct areas in Figure 11c. These two highlighted regions are impacted by the throats affected by the added channel pores. These added throats can be categorized as the channel throats of an area-equivalent radius of around 600 μm and the reconnected channel pore to its neighboring pore throats of an area-equivalent radius between about 250 μm to 400 μm (the minimal area-equivalent radius of the reconnected throat is larger than 250 μm as assumed in the channel modeling). Considering the throat radius distribution, we can explain the behavior of the curves in Figure 11a. The flow in the channeled network in the *x*-axis direction (vertical to the *y*-axis channel direction) shows a sharp increase of saturation from almost 0% to 50% at about 250 μm throat area-equivalent radius. It is in agreement with the reconnected throats region in Figure 11c, since the flow rapidly propagates most of the void space when the capillary pressure is high enough to pass the reconnected throats (250~400 μm radius) and when the channel throats involved in the network are completely connected to the inlet surface. It is a sharp increase since once the invading fluid reaches the first microchannel in the depth of the propagation direction, the pressure currently is high enough to push the fluid to all of the microchannels, involving the micropores in the fluid paths between the microchannels, which takes about 50% of the void space.

The curve of the channeled network in the *y*-axis direction can be also explained considering distribution plot Figure 11c similarly since the microchannels are filled at the beginning and reach about 20% saturation. The saturation curve then smoothly develops and turns identical to the curve of the channeled *x*-axis direction when all of the channel pores and the pores affected by the reconnected throats are filled, and the remaining unfilled pore network is almost identical.

#### 3.2.2. Relative Permeability

**Figure 12:**
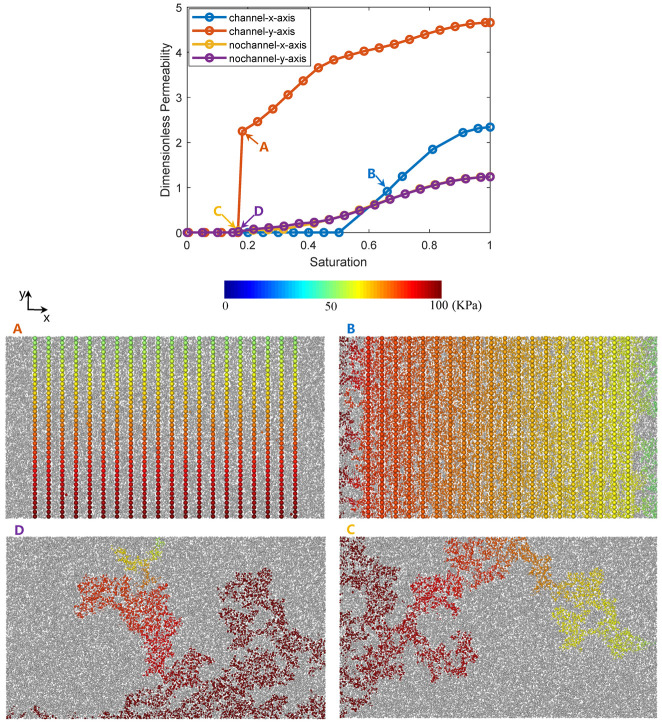
Drainage relative permeability curves and 4 chosen pressure fields (**A**–**D**) in pore networks with (**A**,**B**) and without (**C**,**D**) microchannels.

We finally investigate the so-called drainage process in terms of drainage relative permeability of the water transport with and without microchannels (Figure 12). To obtain the permeability, an arbitrary pressure difference is added between the inlet and outlet boundary (100 KPa and 50 KPa for the inlet and outlet in our case), with the other four lateral surfaces set as the no flux boundaries, and the initial pressures of other pores are assigned as 0 Pa. The dimensionless permeability is then calculated at every 0.05 saturation interval. Note that the dimensionless permeability instead of the relative permeability is chosen for quantitative comparison, and the pressure values used at the boundaries are somewhat arbitrary for the calculation of permeability but not the real boundary pressure values at a certain saturation.

The dimensionless permeability curves in Figure 12 show that the water flow in the channel direction (*y*-axis) predicts the highest dimensionless absolute permeability value (the water phase permeability at 100% water saturation) of about 4.7. The water flow in the direction vertical to the channel direction also predicted a high absolute permeability value of about 2.3. The curves in the *x-* and *y*-axis of the initial network without channel are almost identical and reach about 1.2 absolute permeability. This value (1.2) is in good agreement with the formerly simulated values (between 1 and 1.3) in Figure 9.

We then specifically choose and show 4 pressure fields noted in Figure 12A–D. The colored balls indicate the invaded pores with their pressure values varied by color, and the grey balls show the pores that are not invaded yet. To facilitate graphic rendering, the throats are hidden. Figure 12A shows the breakthrough moment of the flow with microchannels in the *y*-axis direction. As expected, the microchannels provide the highways for fluid to pass through and result in a sharp increase of the permeability curve. After the breakthrough moment, the remaining uninvaded pores of smaller pore sizes and larger flow resistance beside the microchannels will be gradually invaded as the pressure gradient increases. Therefore, the microchannel can provide the highway for the flow path of the reactant, while the rest of the network with pore sizes less than one or several orders of magnitude provide a higher flow resistance and a higher specific surface, and contribute to better support for reaction. In a word, the coupling of multiscale structures results in an ideal multiscale system for the multifunction purpose.

Figure 12B shows the flow path and the pressure distribution of the channeled flow in the *x*-axis direction, which is vertical to the channel arrangement orientation. Compared with Figure 10C,D, and the dimensionless permeability curves we can detect that the occurrence of the breakthrough saturation moment is postponed due to the microchannels, and the fluid is more evenly distributed than without microchannels, since each channel along the flow direction provide a new equal start for the water penetration, and finally promote the absolute permeability compared to the scenario without microchannels. It is noted that we did not provide the permeability value at each saturation step. This is because we detected that the solving of the mass conservation of the linear system fails sometimes, yielding wrong pressure values below the minimal value 50 KPa and resulting in a too-high deviation between the inlet and outlet net fluxes.

Finally, Figure 12C,D shows the breakthrough moment in the *x*-axis and *y*-axis directions at the macro length scale without microchannels. We can see that the flow path is very complex due to the random distribution of pore sizes. Therefore, compared with Figure 12A,B, we can conclude that the microchannels can facilitate the foreseen and optimization of the flow behavior.

## 4. Conclusions

In this work, we propose a systematic methodology to upscale the pore network modeling from the pore scale to the macroscale using the stochastic method. To honor the structural property of the original network, a repeat sampling strategy was applied by copying the structural properties of the elements of the original network to the generated elements of the virtual network. We then mimicked the straight-line channel on the surface of the macroscopic network by removing the channel occupied network elements and refilling the remaining space with new channel pores and reconnecting the throat accordingly to preserve the network connectivity, and finally to generate a macroscopic pore network with cross-scale pore sizes that vary more than an order of magnitude (0.08~1 mm). We investigated the two-phase transport properties with and without microchannels. Compared with the network without microchannels, the network with channels shows the enhanced permeability both of the flow in the parallel and vertical directions to the microchannel. We found that the integration of multiscale structure can proactively achieve the designed high performance and multifunctionality. We also detected that the propagation path in the network with microchannels is more even and predictable. These make our modeling framework a promising methodology that is extendable for considerable applications concerning the performance of an optimization-oriented structure design.

## Figures and Tables

**Table 1:** Pore network simplification, the number of reduced pore elements (pores and throats) at each step.

Porosity	48%		61%		68%		80%		90%	
	Throat	Pore	Throat	Pore	Throat	Pore	Throat	Pore	Throat	Pore
initial	57,995	9847	44,525	7474	30,708	4874	16,617	2309	5300	631
threshold	−30,213	−27	−28,502	−231	−19,744	−342	−10,465	−284	−4014	−191
throat merge	−2533		−1025		-922		−920		−243	
branch prune	−376	−377	−958	−965	−774	−787	−319	−325	−72	−73

## Data Availability

The data presented in this study are available on request from the corresponding author.
